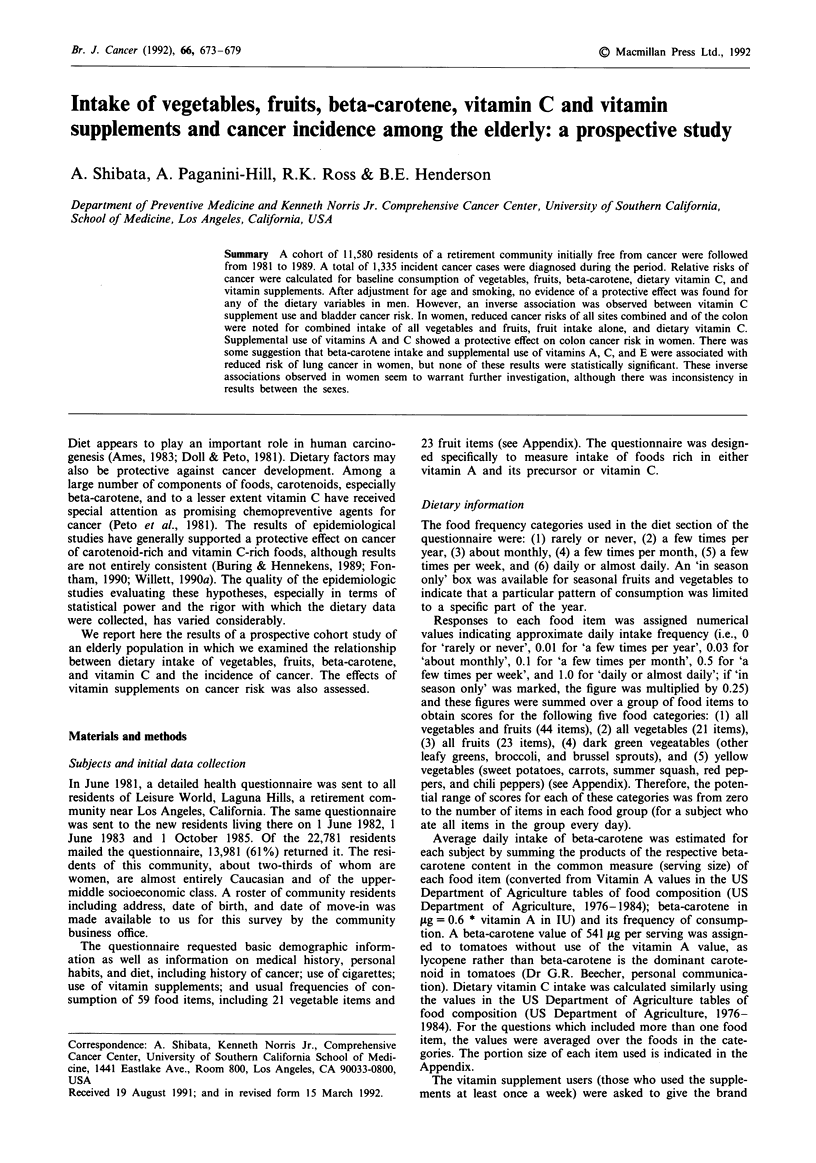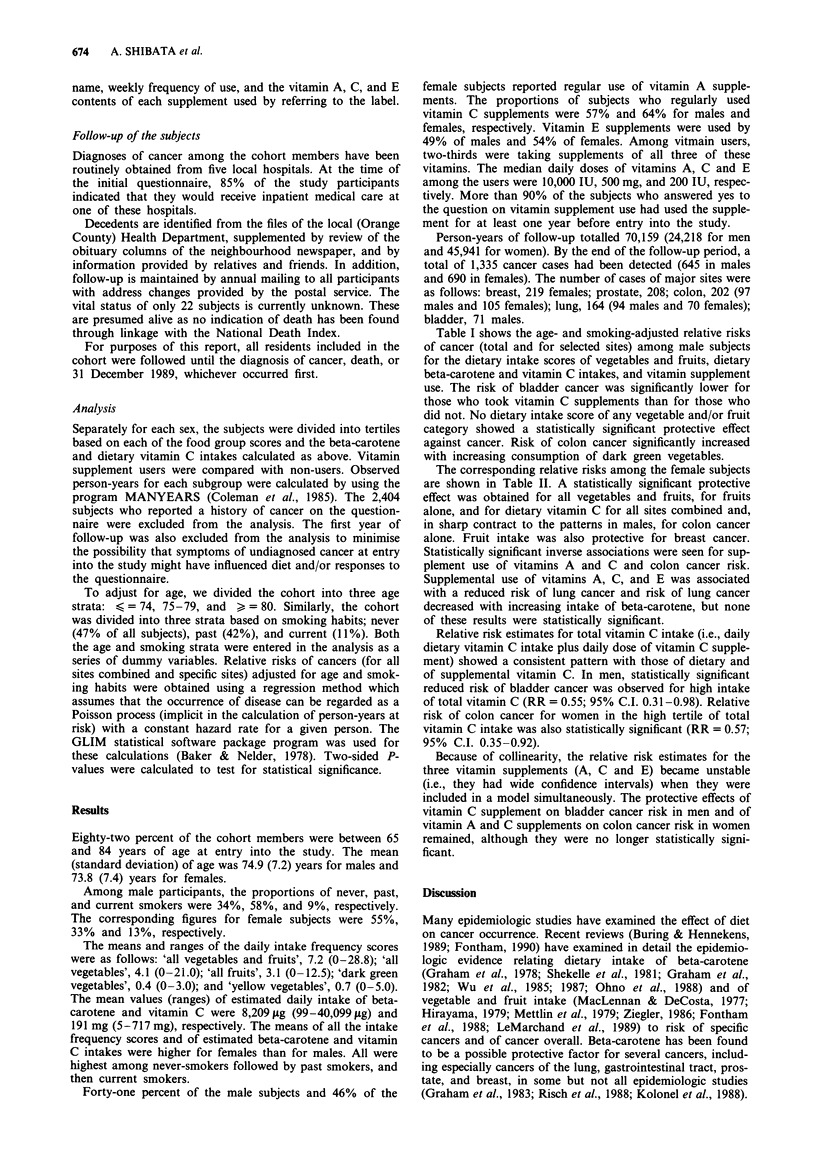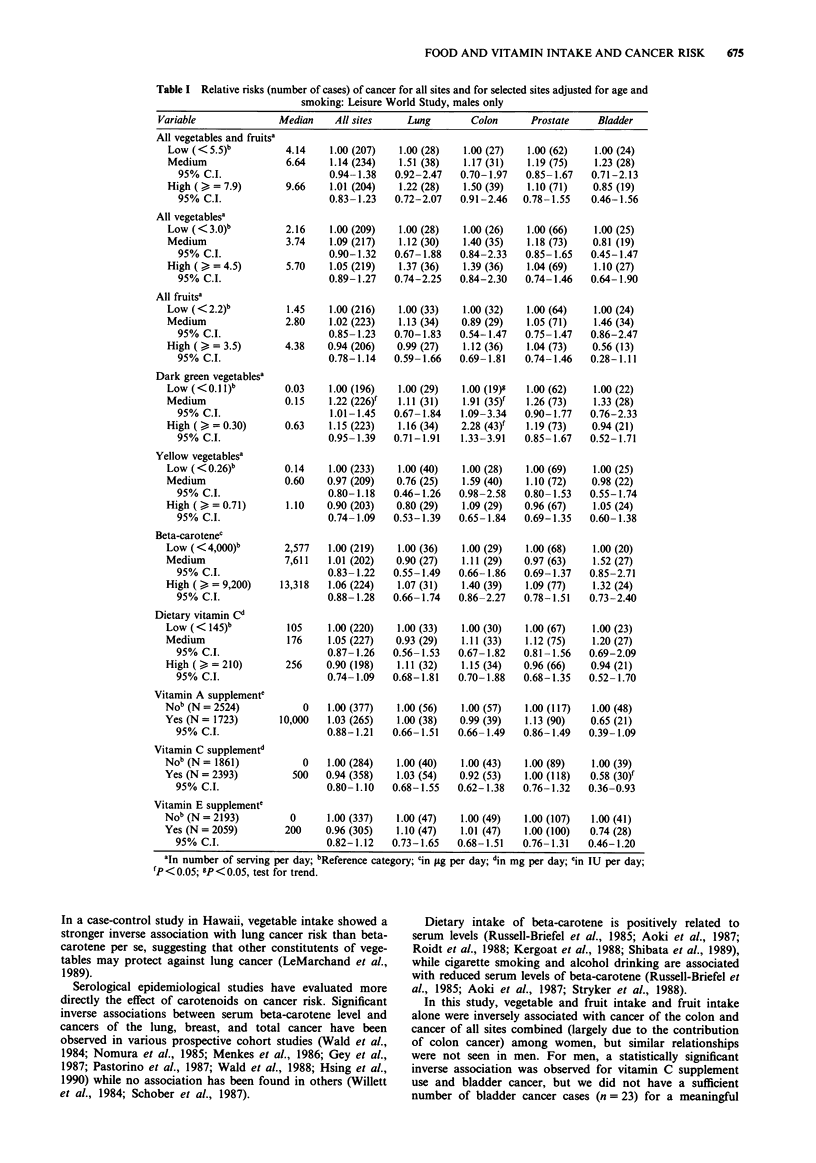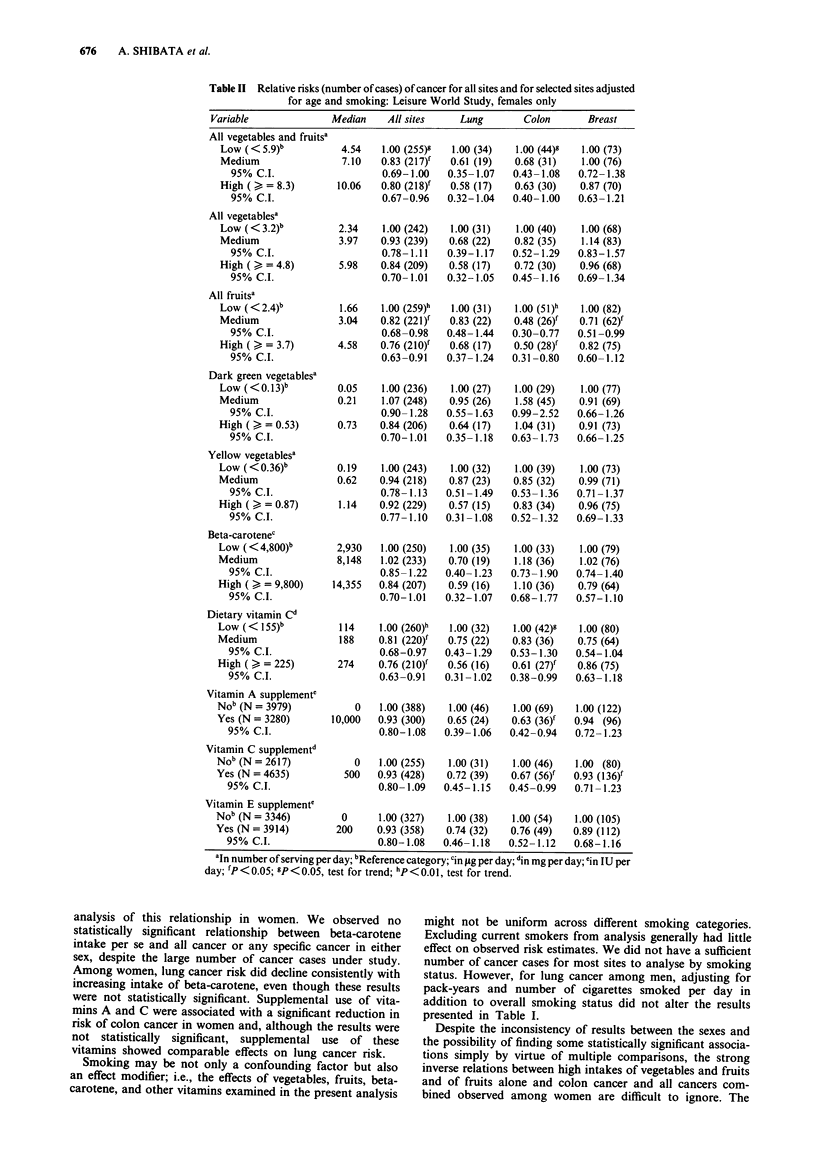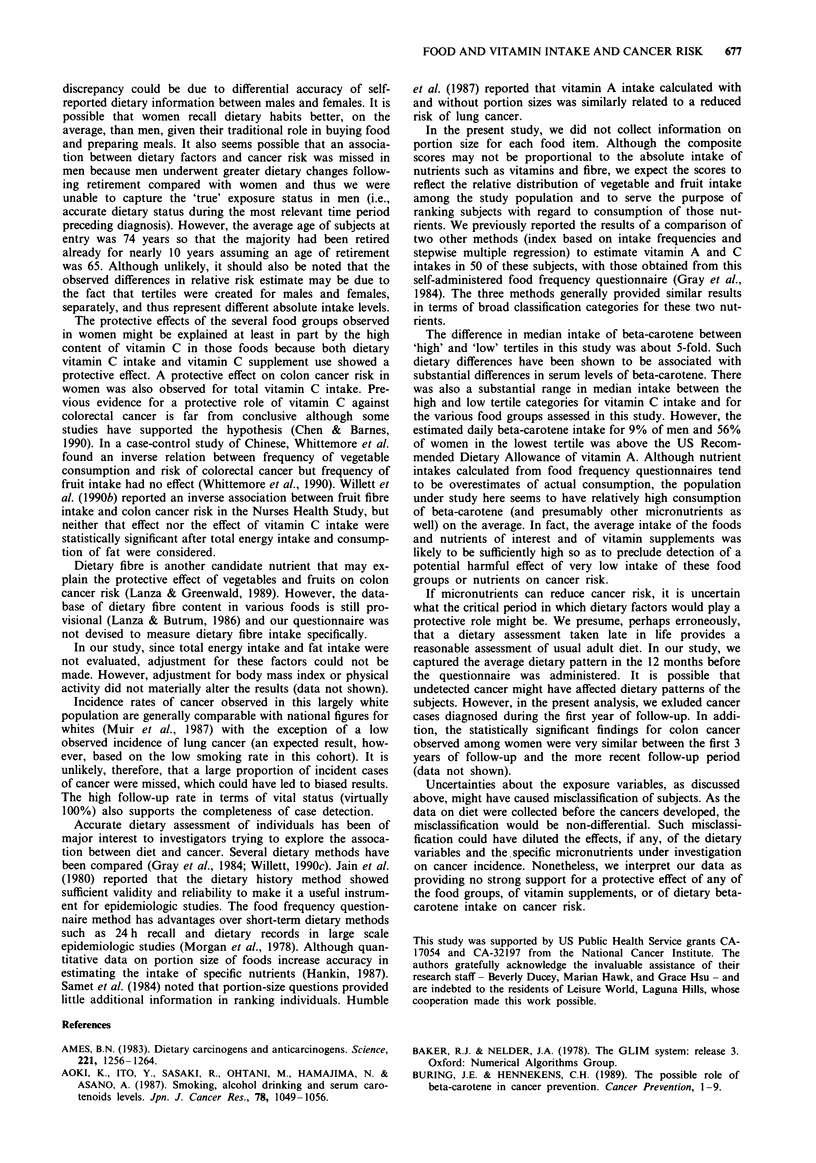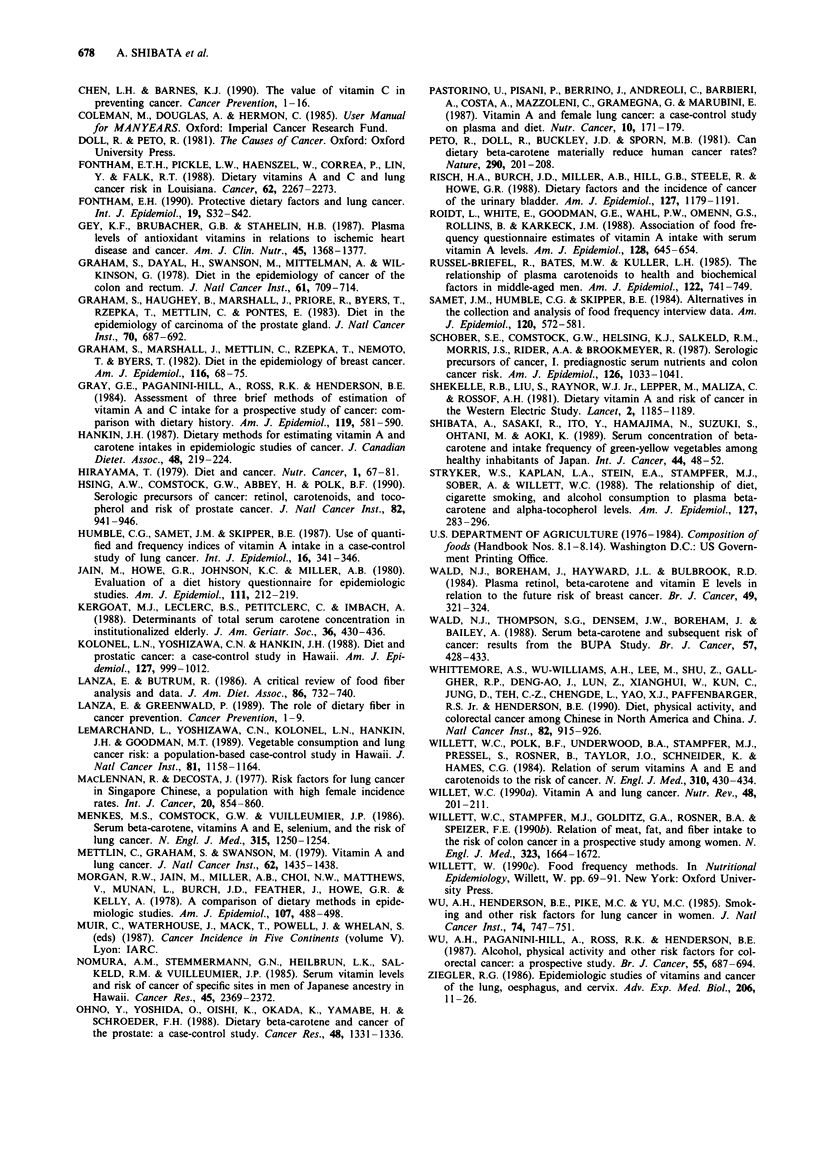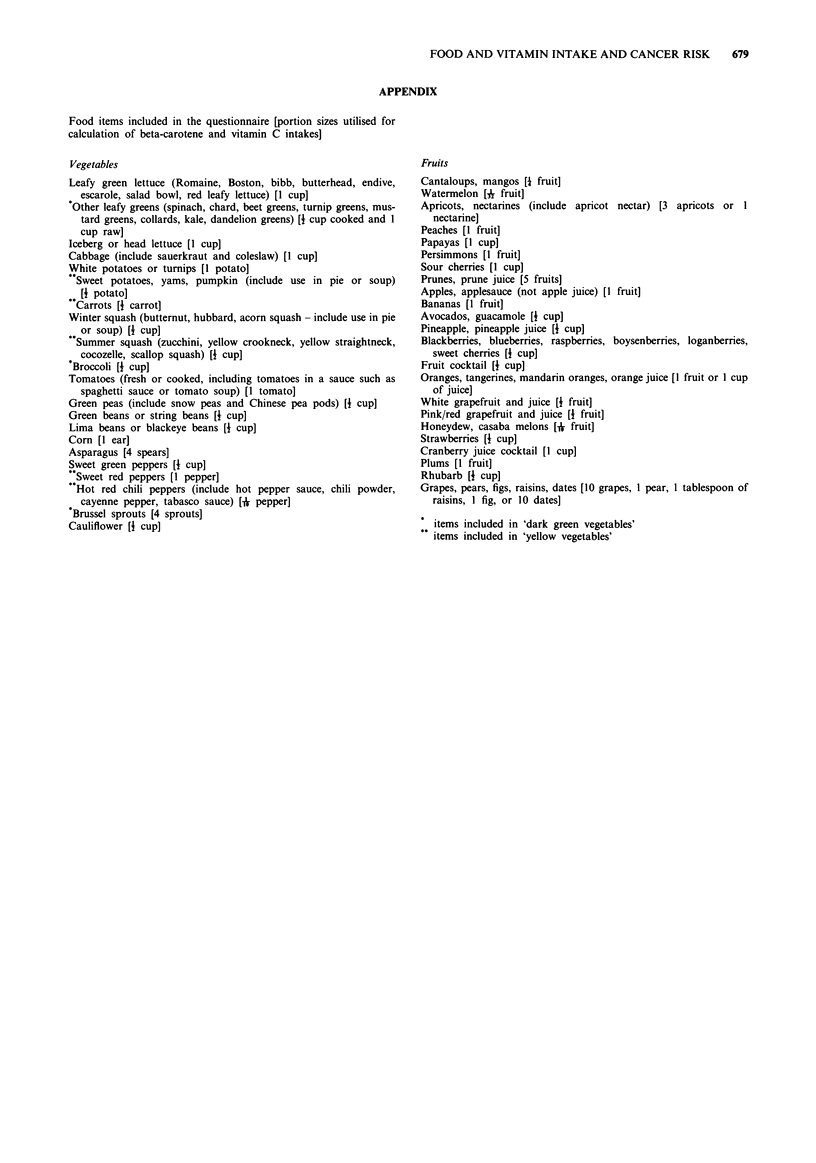# Intake of vegetables, fruits, beta-carotene, vitamin C and vitamin supplements and cancer incidence among the elderly: a prospective study.

**DOI:** 10.1038/bjc.1992.336

**Published:** 1992-10

**Authors:** A. Shibata, A. Paganini-Hill, R. K. Ross, B. E. Henderson

**Affiliations:** Department of Preventive Medicine, University of Southern California, School of Medicine, Los Angeles 90033-0800.

## Abstract

A cohort of 11,580 residents of a retirement community initially free from cancer were followed from 1981 to 1989. A total of 1,335 incident cancer cases were diagnosed during the period. Relative risks of cancer were calculated for baseline consumption of vegetables, fruits, beta-carotene, dietary vitamin C, and vitamin supplements. After adjustment for age and smoking, no evidence of a protective effect was found for any of the dietary variables in men. However, an inverse association was observed between vitamin C supplement use and bladder cancer risk. In women, reduced cancer risks of all sites combined and of the colon were noted for combined intake of all vegetables and fruits, fruit intake alone, and dietary vitamin C. Supplemental use of vitamins A and C showed a protective effect on colon cancer risk in women. There was some suggestion that beta-carotene intake and supplemental use of vitamin A, C, and E were associated with reduced risk of lung cancer in women, but none of these results were statistically significant. These inverse associations observed in women seem to warrant further investigation, although there was inconsistency in results between the sexes.


Br. J. Cancer (1992), 66, 673-679 © Macmillan Press Ltd., 1992

### Intake of vegetables, fruits, beta-carotene, vitamin C and vitamin supplements and cancer incidence among the elderly: a prospective study

A. Shibata, A. Paganini-Hill, R.K. Ross & B.E. Henderson

*Department of Preventive Medicine and Kenneth Norris Jr. Comprehensive Cancer Center, University of Southern California, School of Medicine, Los Angeles, California, USA*

**Summary** A cohort of 11,580 residents of a retirement community initially free from cancer were followed from 1981 to 1989. A total of 1,335 incident cancer cases were diagnosed during the period. Relative risks of cancer were calculated for baseline consumption of vegetables, fruits, beta-carotene, dietary vitamin C, and vitamin supplements. After adjustment for age and smoking, no evidence of a protective effect was found for any of the dietary variables in men. However, an inverse association was observed between vitamin C supplement use and bladder cancer risk. In women, reduced cancer risks of all sites combined and of the colon were noted for combined intake of all vegetables and fruits, fruit intake alone, and dietary vitamin C. Supplemental use of vitamins A and C showed a protective effect on colon cancer risk in women. There was some suggestion that beta-carotene intake and supplemental use of vitamins A, C, and E were associated with reduced risk of lung cancer in women, but none of these results were statistically significant. These inverse associations observed in women seem to warrant further investigation, although there was inconsistency in results between the sexes.

Diet appears to play an important role in human carcinogenesis (Ames, 1983; Doll & Peto, 1981). Dietary factors may also be protective against cancer development. Among a large number of components of foods, carotenoids, especially beta-carotene, and to a lesser extent vitamin C have received special attention as promising chemopreventive agents for cancer (Peto *et al.*, 1981). The results of epidemiological studies have generally supported a protective effect on cancer of carotenoid-rich and vitamin C-rich foods, although results are not entirely consistent (Buring & Hennekens, 1989; Fontham, 1990; Willett, 1990*a*). The quality of the epidemiologic studies evaluating these hypotheses, especially in terms of statistical power and the rigor with which the dietary data were collected, has varied considerably.

We report here the results of a prospective cohort study of an elderly population in which we examined the relationship between dietary intake of vegetables, fruits, beta-carotene, and vitamin C and the incidence of cancer. The effects of vitamin supplements on cancer risk was also assessed.

### Materials and methods

### *Subjects and initial data collection*

In June 1981, a detailed health questionnaire was sent to all residents of Leisure World, Laguna Hills, a retirement community near Los Angeles, California. The same questionnaire was sent to the new residents living there on 1 June 1982, 1 June 1983 and 1 October 1985. Of the 22,781 residents mailed the questionnaire, 13,981 (61%) returned it. The residents of this community, about two-thirds of whom are women, are almost entirely Caucasian and of the upper-middle socioeconomic class. A roster of community residents including address, date of birth, and date of move-in was made available to us for this survey by the community business office.

The questionnaire requested basic demographic information as well as information on medical history, personal habits, and diet, including history of cancer; use of cigarettes; use of vitamin supplements; and usual frequencies of consumption of 59 food items, including 21 vegetable items and 23 fruit items (see Appendix). The questionnaire was designed specifically to measure intake of foods rich in either vitamin A and its precursor or vitamin C.

### *Dietary information*

The food frequency categories used in the diet section of the questionnaire were: (1) rarely or never, (2) a few times per year, (3) about monthly, (4) a few times per month, (5) a few times per week, and (6) daily or almost daily. An 'in season only' box was available for seasonal fruits and vegetables to indicate that a particular pattern of consumption was limited to a specific part of the year.

Responses to each food item was assigned numerical values indicating approximate daily intake frequency (i.e., 0 for 'rarely or never', 0.01 for 'a few times per year', 0.03 for 'about monthly', 0.1 for 'a few times per month', 0.5 for 'a few times per week', and 1.0 for 'daily or almost daily'; if 'in season only' was marked, the figure was multiplied by 0.25) and these figures were summed over a group of food items to obtain scores for the following five food categories: (1) all vegetables and fruits (44 items), (2) all vegetables (21 items), (3) all fruits (23 items), (4) dark green vegeatables (other leafy greens, broccoli, and brussel sprouts), and (5) yellow vegetables (sweet potatoes, carrots, summer squash, red peppers, and chili peppers) (see Appendix). Therefore, the potential range of scores for each of these categories was from zero to the number of items in each food group (for a subject who ate all items in the group every day).

Average daily intake of beta-carotene was estimated for each subject by summing the products of the respective beta-carotene content in the common measure (serving size) of each food item (converted from Vitamin A values in the US Department of Agriculture tables of food composition (US Department of Agriculture, 1976-1984); beta-carotene in $\mu g = 0.6 *$ vitamin A in IU) and its frequency of consumption. A beta-carotene value of 541 $\mu g$ per serving was assigned to tomatoes without use of the vitamin A value, as lycopene rather than beta-carotene is the dominant carotenoid in tomatoes (Dr G.R. Beecher, personal communication). Dietary vitamin C intake was calculated similarly using the values in the US Department of Agriculture tables of food composition (US Department of Agriculture, 1976-1984). For the questions which included more than one food item, the values were averaged over the foods in the categories. The portion size of each item used is indicated in the Appendix.

The vitamin supplement users (those who used the supplements at least once a week) were asked to give the brand

Correspondence: A. Shibata, Kenneth Norris Jr., Comprehensive Cancer Center, University of Southern California School of Medicine, 1441 Eastlake Ave., Room 800, Los Angeles, CA 90033-0800, USA
Received 19 August 1991; and in revised form 15 March 1992.

name, weekly frequency of use, and the vitamin A, C, and E contents of each supplement used by referring to the label.

*Follow-up of the subjects*

Diagnoses of cancer among the cohort members have been routinely obtained from five local hospitals. At the time of the initial questionnaire, 85% of the study participants indicated that they would receive inpatient medical care at one of these hospitals.

Decedents are identified from the files of the local (Orange County) Health Department, supplemented by review of the obituary columns of the neighbourhood newspaper, and by information provided by relatives and friends. In addition, follow-up is maintained by annual mailing to all participants with address changes provided by the postal service. The vital status of only 22 subjects is currently unknown. These are presumed alive as no indication of death has been found through linkage with the National Death Index.

For purposes of this report, all residents included in the cohort were followed until the diagnosis of cancer, death, or 31 December 1989, whichever occurred first.

*Analysis*

Separately for each sex, the subjects were divided into tertiles based on each of the food group scores and the beta-carotene and dietary vitamin C intakes calculated as above. Vitamin supplement users were compared with non-users. Observed person-years for each subgroup were calculated by using the program MANYEARS (Coleman *et al.*, 1985). The 2,404 subjects who reported a history of cancer on the questionnaire were excluded from the analysis. The first year of follow-up was also excluded from the analysis to minimise the possibility that symptoms of undiagnosed cancer at entry into the study might have influenced diet and/or responses to the questionnaire.

To adjust for age, we divided the cohort into three age strata: $\leqslant = 74$, 75–79, and $\geqslant = 80$. Similarly, the cohort was divided into three strata based on smoking habits; never (47% of all subjects), past (42%), and current (11%). Both the age and smoking strata were entered in the analysis as a series of dummy variables. Relative risks of cancers (for all sites combined and specific sites) adjusted for age and smoking habits were obtained using a regression method which assumes that the occurrence of disease can be regarded as a Poisson process (implicit in the calculation of person-years at risk) with a constant hazard rate for a given person. The GLIM statistical software package program was used for these calculations (Baker & Nelder, 1978). Two-sided $P$-values were calculated to test for statistical significance.

### Results

Eighty-two percent of the cohort members were between 65 and 84 years of age at entry into the study. The mean (standard deviation) of age was 74.9 (7.2) years for males and 73.8 (7.4) years for females.

Among male participants, the proportions of never, past, and current smokers were 34%, 58%, and 9%, respectively. The corresponding figures for female subjects were 55%, 33% and 13%, respectively.

The means and ranges of the daily intake frequency scores were as follows: 'all vegetables and fruits', 7.2 (0–28.8); 'all vegetables', 4.1 (0–21.0); 'all fruits', 3.1 (0–12.5); 'dark green vegetables', 0.4 (0–3.0); and 'yellow vegetables', 0.7 (0–5.0). The mean values (ranges) of estimated daily intake of beta-carotene and vitamin C were 8,209 μg (99–40,099 μg) and 191 mg (5–717 mg), respectively. The means of all the intake frequency scores and of estimated beta-carotene and vitamin C intakes were higher for females than for males. All were highest among never-smokers followed by past smokers, and then current smokers.

Forty-one percent of the male subjects and 46% of the female subjects reported regular use of vitamin A supplements. The proportions of subjects who regularly used vitamin C supplements were 57% and 64% for males and females, respectively. Vitamin E supplements were used by 49% of males and 54% of females. Among vitmain users, two-thirds were taking supplements of all three of these vitamins. The median daily doses of vitamins A, C and E among the users were 10,000 IU, 500 mg, and 200 IU, respectively. More than 90% of the subjects who answered yes to the question on vitamin supplement use had used the supplement for at least one year before entry into the study.

Person-years of follow-up totalled 70,159 (24,218 for men and 45,941 for women). By the end of the follow-up period, a total of 1,335 cancer cases had been detected (645 in males and 690 in females). The number of cases of major sites were as follows: breast, 219 females; prostate, 208; colon, 202 (97 males and 105 females); lung, 164 (94 males and 70 females); bladder, 71 males.

Table I shows the age- and smoking-adjusted relative risks of cancer (total and for selected sites) among male subjects for the dietary intake scores of vegetables and fruits, dietary beta-carotene and vitamin C intakes, and vitamin supplement use. The risk of bladder cancer was significantly lower for those who took vitamin C supplements than for those who did not. No dietary intake score of any vegetable and/or fruit category showed a statistically significant protective effect against cancer. Risk of colon cancer significantly increased with increasing consumption of dark green vegetables.

The corresponding relative risks among the female subjects are shown in Table II. A statistically significant protective effect was obtained for all vegetables and fruits, for fruits alone, and for dietary vitamin C for all sites combined and, in sharp contract to the patterns in males, for colon cancer alone. Fruit intake was also protective for breast cancer. Statistically significant inverse associations were seen for supplement use of vitamins A and C and colon cancer risk. Supplemental use of vitamins A, C, and E was associated with a reduced risk of lung cancer and risk of lung cancer decreased with increasing intake of beta-carotene, but none of these results were statistically significant.

Relative risk estimates for total vitamin C intake (i.e., daily dietary vitamin C intake plus daily dose of vitamin C supplement) showed a consistent pattern with those of dietary and of supplemental vitamin C. In men, statistically significant reduced risk of bladder cancer was observed for high intake of total vitamin C (RR = 0.55; 95% C.I. 0.31–0.98). Relative risk of colon cancer for women in the high tertile of total vitamin C intake was also statistically significant (RR = 0.57; 95% C.I. 0.35–0.92).

Because of collinearity, the relative risk estimates for the three vitamin supplements (A, C and E) became unstable (i.e., they had wide confidence intervals) when they were included in a model simultaneously. The protective effects of vitamin C supplement on bladder cancer risk in men and of vitamin A and C supplements on colon cancer risk in women remained, although they were no longer statistically significant.

### Discussion

Many epidemiologic studies have examined the effect of diet on cancer occurrence. Recent reviews (Buring & Hennekens, 1989; Fontham, 1990) have examined in detail the epidemiologic evidence relating dietary intake of beta-carotene (Graham *et al.*, 1978; Shekelle *et al.*, 1981; Graham *et al.*, 1982; Wu *et al.*, 1985; 1987; Ohno *et al.*, 1988) and of vegetable and fruit intake (MacLennan & DeCosta, 1977; Hirayama, 1979; Mettlin *et al.*, 1979; Ziegler, 1986; Fontham *et al.*, 1988; LeMarchand *et al.*, 1989) to risk of specific cancers and of cancer overall. Beta-carotene has been found to be a possible protective factor for several cancers, including especially cancers of the lung, gastrointestinal tract, prostate, and breast, in some but not all epidemiologic studies (Graham *et al.*, 1983; Risch *et al.*, 1988; Kolonel *et al.*, 1988).

**Table I** Relative risks (number of cases) of cancer for all sites and for selected sites adjusted for age and smoking: Leisure World Study, males only

| *Variable* | *Median* | *All sites* | *Lung* | *Colon* | *Prostate* | *Bladder* |
|---|---|---|---|---|---|---|
| All vegetables and fruits[a] | | | | | | |
| Low (<5.5)[b] | 4.14 | 1.00 (207) | 1.00 (28) | 1.00 (27) | 1.00 (62) | 1.00 (24) |
| Medium | 6.64 | 1.14 (234) | 1.51 (38) | 1.17 (31) | 1.19 (75) | 1.23 (28) |
| 95% C.I. | | 0.94–1.38 | 0.92–2.47 | 0.70–1.97 | 0.85–1.67 | 0.71–2.13 |
| High (⩾ = 7.9) | 9.66 | 1.01 (204) | 1.22 (28) | 1.50 (39) | 1.10 (71) | 0.85 (19) |
| 95% C.I. | | 0.83–1.23 | 0.72–2.07 | 0.91–2.46 | 0.78–1.55 | 0.46–1.56 |
| All vegetables[a] | | | | | | |
| Low (<3.0)[b] | 2.16 | 1.00 (209) | 1.00 (28) | 1.00 (26) | 1.00 (66) | 1.00 (25) |
| Medium | 3.74 | 1.09 (217) | 1.12 (30) | 1.40 (35) | 1.18 (73) | 0.81 (19) |
| 95% C.I. | | 0.90–1.32 | 0.67–1.88 | 0.84–2.33 | 0.85–1.65 | 0.45–1.47 |
| High (⩾ = 4.5) | 5.70 | 1.05 (219) | 1.37 (36) | 1.39 (36) | 1.04 (69) | 1.10 (27) |
| 95% C.I. | | 0.89–1.27 | 0.74–2.25 | 0.84–2.30 | 0.74–1.46 | 0.64–1.90 |
| All fruits[a] | | | | | | |
| Low (<2.2)[b] | 1.45 | 1.00 (216) | 1.00 (33) | 1.00 (32) | 1.00 (64) | 1.00 (24) |
| Medium | 2.80 | 1.02 (223) | 1.13 (34) | 0.89 (29) | 1.05 (71) | 1.46 (34) |
| 95% C.I. | | 0.85–1.23 | 0.70–1.83 | 0.54–1.47 | 0.75–1.47 | 0.86–2.47 |
| High (⩾ = 3.5) | 4.38 | 0.94 (206) | 0.99 (27) | 1.12 (36) | 1.04 (73) | 0.56 (13) |
| 95% C.I. | | 0.78–1.14 | 0.59–1.66 | 0.69–1.81 | 0.74–1.46 | 0.28–1.11 |
| Dark green vegetables[a] | | | | | | |
| Low (<0.11)[b] | 0.03 | 1.00 (196) | 1.00 (29) | 1.00 (19)[g] | 1.00 (62) | 1.00 (22) |
| Medium | 0.15 | 1.22 (226)[f] | 1.11 (31) | 1.91 (35)[f] | 1.26 (73) | 1.33 (28) |
| 95% C.I. | | 1.01–1.45 | 0.67–1.84 | 1.09–3.34 | 0.90–1.77 | 0.76–2.33 |
| High (⩾ = 0.30) | 0.63 | 1.15 (223) | 1.16 (34) | 2.28 (43)[f] | 1.19 (73) | 0.94 (21) |
| 95% C.I. | | 0.95–1.39 | 0.71–1.91 | 1.33–3.91 | 0.85–1.67 | 0.52–1.71 |
| Yellow vegetables[a] | | | | | | |
| Low (<0.26)[b] | 0.14 | 1.00 (233) | 1.00 (40) | 1.00 (28) | 1.00 (69) | 1.00 (25) |
| Medium | 0.60 | 0.97 (209) | 0.76 (25) | 1.59 (40) | 1.10 (72) | 0.98 (22) |
| 95% C.I. | | 0.80–1.18 | 0.46–1.26 | 0.98–2.58 | 0.80–1.53 | 0.55–1.74 |
| High (⩾ = 0.71) | 1.10 | 0.90 (203) | 0.80 (29) | 1.09 (29) | 0.96 (67) | 1.05 (24) |
| 95% C.I. | | 0.74–1.09 | 0.53–1.39 | 0.65–1.84 | 0.69–1.35 | 0.60–1.38 |
| Beta-carotene[c] | | | | | | |
| Low (<4,000)[b] | 2,577 | 1.00 (219) | 1.00 (36) | 1.00 (29) | 1.00 (68) | 1.00 (20) |
| Medium | 7,611 | 1.01 (202) | 0.90 (27) | 1.11 (29) | 0.97 (63) | 1.52 (27) |
| 95% C.I. | | 0.83–1.22 | 0.55–1.49 | 0.66–1.86 | 0.69–1.37 | 0.85–2.71 |
| High (⩾ = 9,200) | 13,318 | 1.06 (224) | 1.07 (31) | 1.40 (39) | 1.09 (77) | 1.32 (24) |
| 95% C.I. | | 0.88–1.28 | 0.66–1.74 | 0.86–2.27 | 0.78–1.51 | 0.73–2.40 |
| Dietary vitamin C[d] | | | | | | |
| Low (<145)[b] | 105 | 1.00 (220) | 1.00 (33) | 1.00 (30) | 1.00 (67) | 1.00 (23) |
| Medium | 176 | 1.05 (227) | 0.93 (29) | 1.11 (33) | 1.12 (75) | 1.20 (27) |
| 95% C.I. | | 0.87–1.26 | 0.56–1.53 | 0.67–1.82 | 0.81–1.56 | 0.69–2.09 |
| High (⩾ = 210) | 256 | 0.90 (198) | 1.11 (32) | 1.15 (34) | 0.96 (66) | 0.94 (21) |
| 95% C.I. | | 0.74–1.09 | 0.68–1.81 | 0.70–1.88 | 0.68–1.35 | 0.52–1.70 |
| Vitamin A supplement[e] | | | | | | |
| No[b] (N = 2524) | 0 | 1.00 (377) | 1.00 (56) | 1.00 (57) | 1.00 (117) | 1.00 (48) |
| Yes (N = 1723) | 10,000 | 1.03 (265) | 1.00 (38) | 0.99 (39) | 1.13 (90) | 0.65 (21) |
| 95% C.I. | | 0.88–1.21 | 0.66–1.51 | 0.66–1.49 | 0.86–1.49 | 0.39–1.09 |
| Vitamin C supplement[d] | | | | | | |
| No[b] (N = 1861) | 0 | 1.00 (284) | 1.00 (40) | 1.00 (43) | 1.00 (89) | 1.00 (39) |
| Yes (N = 2393) | 500 | 0.94 (358) | 1.03 (54) | 0.92 (53) | 1.00 (118) | 0.58 (30)[f] |
| 95% C.I. | | 0.80–1.10 | 0.68–1.55 | 0.62–1.38 | 0.76–1.32 | 0.36–0.93 |
| Vitamin E supplement[e] | | | | | | |
| No[b] (N = 2193) | 0 | 1.00 (337) | 1.00 (47) | 1.00 (49) | 1.00 (107) | 1.00 (41) |
| Yes (N = 2059) | 200 | 0.96 (305) | 1.10 (47) | 1.01 (47) | 1.00 (100) | 0.74 (28) |
| 95% C.I. | | 0.82–1.12 | 0.73–1.65 | 0.68–1.51 | 0.76–1.31 | 0.46–1.20 |

[a]In number of serving per day; [b]Reference category; [c]in μg per day; [d]in mg per day; [e]in IU per day; [f]$P<0.05$; [g]$P<0.05$, test for trend.

In a case-control study in Hawaii, vegetable intake showed a stronger inverse association with lung cancer risk than beta-carotene per se, suggesting that other constitutents of vegetables may protect against lung cancer (LeMarchand *et al.*, 1989).

Serological epidemiological studies have evaluated more directly the effect of carotenoids on cancer risk. Significant inverse associations between serum beta-carotene level and cancers of the lung, breast, and total cancer have been observed in various prospective cohort studies (Wald *et al.*, 1984; Nomura *et al.*, 1985; Menkes *et al.*, 1986; Gey *et al.*, 1987; Pastorino *et al.*, 1987; Wald *et al.*, 1988; Hsing *et al.*, 1990) while no association has been found in others (Willett *et al.*, 1984; Schober *et al.*, 1987).

Dietary intake of beta-carotene is positively related to serum levels (Russell-Briefel *et al.*, 1985; Aoki *et al.*, 1987; Roidt *et al.*, 1988; Kergoat *et al.*, 1988; Shibata *et al.*, 1989), while cigarette smoking and alcohol drinking are associated with reduced serum levels of beta-carotene (Russell-Briefel *et al.*, 1985; Aoki *et al.*, 1987; Stryker *et al.*, 1988).

In this study, vegetable and fruit intake and fruit intake alone were inversely associated with cancer of the colon and cancer of all sites combined (largely due to the contribution of colon cancer) among women, but similar relationships were not seen in men. For men, a statistically significant inverse association was observed for vitamin C supplement use and bladder cancer, but we did not have a sufficient number of bladder cancer cases ($n = 23$) for a meaningful

**Table II** Relative risks (number of cases) of cancer for all sites and for selected sites adjusted for age and smoking: Leisure World Study, females only

| *Variable* | *Median* | *All sites* | *Lung* | *Colon* | *Breast* |
|---|---|---|---|---|---|
| All vegetables and fruits[a] | | | | | |
| Low (<5.9)[b] | 4.54 | 1.00 (255)[g] | 1.00 (34) | 1.00 (44)[g] | 1.00 (73) |
| Medium | 7.10 | 0.83 (217)[f] | 0.61 (19) | 0.68 (31) | 1.00 (76) |
| 95% C.I. | | 0.69-1.00 | 0.35-1.07 | 0.43-1.08 | 0.72-1.38 |
| High (⩾ = 8.3) | 10.06 | 0.80 (218)[f] | 0.58 (17) | 0.63 (30) | 0.87 (70) |
| 95% C.I. | | 0.67-0.96 | 0.32-1.04 | 0.40-1.00 | 0.63-1.21 |
| All vegetables[a] | | | | | |
| Low (<3.2)[b] | 2.34 | 1.00 (242) | 1.00 (31) | 1.00 (40) | 1.00 (68) |
| Medium | 3.97 | 0.93 (239) | 0.68 (22) | 0.82 (35) | 1.14 (83) |
| 95% C.I. | | 0.78-1.11 | 0.39-1.17 | 0.52-1.29 | 0.83-1.57 |
| High (⩾ = 4.8) | 5.98 | 0.84 (209) | 0.58 (17) | 0.72 (30) | 0.96 (68) |
| 95% C.I. | | 0.70-1.01 | 0.32-1.05 | 0.45-1.16 | 0.69-1.34 |
| All fruits[a] | | | | | |
| Low (<2.4)[b] | 1.66 | 1.00 (259)[h] | 1.00 (31) | 1.00 (51)[h] | 1.00 (82) |
| Medium | 3.04 | 0.82 (221)[f] | 0.83 (22) | 0.48 (26)[f] | 0.71 (62)[f] |
| 95% C.I. | | 0.68-0.98 | 0.48-1.44 | 0.30-0.77 | 0.51-0.99 |
| High (⩾ = 3.7) | 4.58 | 0.76 (210)[f] | 0.68 (17) | 0.50 (28)[f] | 0.82 (75) |
| 95% C.I. | | 0.63-0.91 | 0.37-1.24 | 0.31-0.80 | 0.60-1.12 |
| Dark green vegetables[a] | | | | | |
| Low (<0.13)[b] | 0.05 | 1.00 (236) | 1.00 (27) | 1.00 (29) | 1.00 (77) |
| Medium | 0.21 | 1.07 (248) | 0.95 (26) | 1.58 (45) | 0.91 (69) |
| 95% C.I. | | 0.90-1.28 | 0.55-1.63 | 0.99-2.52 | 0.66-1.26 |
| High (⩾ = 0.53) | 0.73 | 0.84 (206) | 0.64 (17) | 1.04 (31) | 0.91 (73) |
| 95% C.I. | | 0.70-1.01 | 0.35-1.18 | 0.63-1.73 | 0.66-1.25 |
| Yellow vegetables[a] | | | | | |
| Low (<0.36)[b] | 0.19 | 1.00 (243) | 1.00 (32) | 1.00 (39) | 1.00 (73) |
| Medium | 0.62 | 0.94 (218) | 0.87 (23) | 0.85 (32) | 0.99 (71) |
| 95% C.I. | | 0.78-1.13 | 0.51-1.49 | 0.53-1.36 | 0.71-1.37 |
| High (⩾ = 0.87) | 1.14 | 0.92 (229) | 0.57 (15) | 0.83 (34) | 0.96 (75) |
| 95% C.I. | | 0.77-1.10 | 0.31-1.08 | 0.52-1.32 | 0.69-1.33 |
| Beta-carotene[c] | | | | | |
| Low (<4,800)[b] | 2,930 | 1.00 (250) | 1.00 (35) | 1.00 (33) | 1.00 (79) |
| Medium | 8,148 | 1.02 (233) | 0.70 (19) | 1.18 (36) | 1.02 (76) |
| 95% C.I. | | 0.85-1.22 | 0.40-1.23 | 0.73-1.90 | 0.74-1.40 |
| High (⩾ = 9,800) | 14,355 | 0.84 (207) | 0.59 (16) | 1.10 (36) | 0.79 (64) |
| 95% C.I. | | 0.70-1.01 | 0.32-1.07 | 0.68-1.77 | 0.57-1.10 |
| Dietary vitamin C[d] | | | | | |
| Low (<155)[b] | 114 | 1.00 (260)[h] | 1.00 (32) | 1.00 (42)[g] | 1.00 (80) |
| Medium | 188 | 0.81 (220)[f] | 0.75 (22) | 0.83 (36) | 0.75 (64) |
| 95% C.I. | | 0.68-0.97 | 0.43-1.29 | 0.53-1.30 | 0.54-1.04 |
| High (⩾ = 225) | 274 | 0.76 (210)[f] | 0.56 (16) | 0.61 (27)[f] | 0.86 (75) |
| 95% C.I. | | 0.63-0.91 | 0.31-1.02 | 0.38-0.99 | 0.63-1.18 |
| Vitamin A supplement[e] | | | | | |
| No[b] (N = 3979) | 0 | 1.00 (388) | 1.00 (46) | 1.00 (69) | 1.00 (122) |
| Yes (N = 3280) | 10,000 | 0.93 (300) | 0.65 (24) | 0.63 (36)[f] | 0.94 (96) |
| 95% C.I. | | 0.80-1.08 | 0.39-1.06 | 0.42-0.94 | 0.72-1.23 |
| Vitamin C supplement[d] | | | | | |
| No[b] (N = 2617) | 0 | 1.00 (255) | 1.00 (31) | 1.00 (46) | 1.00 (80) |
| Yes (N = 4635) | 500 | 0.93 (428) | 0.72 (39) | 0.67 (56)[f] | 0.93 (136)[f] |
| 95% C.I. | | 0.80-1.09 | 0.45-1.15 | 0.45-0.99 | 0.71-1.23 |
| Vitamin E supplement[e] | | | | | |
| No[b] (N = 3346) | 0 | 1.00 (327) | 1.00 (38) | 1.00 (54) | 1.00 (105) |
| Yes (N = 3914) | 200 | 0.93 (358) | 0.74 (32) | 0.76 (49) | 0.89 (112) |
| 95% C.I. | | 0.80-1.08 | 0.46-1.18 | 0.52-1.12 | 0.68-1.16 |

[a]In number of serving per day; [b]Reference category; [c]in μg per day; [d]in mg per day; [e]in IU per day; [f]$P<0.05$; [g]$P<0.05$, test for trend; [h]$P<0.01$, test for trend.

analysis of this relationship in women. We observed no statistically significant relationship between beta-carotene intake per se and all cancer or any specific cancer in either sex, despite the large number of cancer cases under study. Among women, lung cancer risk did decline consistently with increasing intake of beta-carotene, even though these results were not statistically significant. Supplemental use of vitamins A and C were associated with a significant reduction in risk of colon cancer in women and, although the results were not statistically significant, supplemental use of these vitamins showed comparable effects on lung cancer risk.

Smoking may be not only a confounding factor but also an effect modifier; i.e., the effects of vegetables, fruits, beta-carotene, and other vitamins examined in the present analysis might not be uniform across different smoking categories. Excluding current smokers from analysis generally had little effect on observed risk estimates. We did not have a sufficient number of cancer cases for most sites to analyse by smoking status. However, for lung cancer among men, adjusting for pack-years and number of cigarettes smoked per day in addition to overall smoking status did not alter the results presented in Table I.

Despite the inconsistency of results between the sexes and the possibility of finding some statistically significant associations simply by virtue of multiple comparisons, the strong inverse relations between high intakes of vegetables and fruits and of fruits alone and colon cancer and all cancers combined observed among women are difficult to ignore. The discrepancy could be due to differential accuracy of self-reported dietary information between males and females. It is possible that women recall dietary habits better, on the average, than men, given their traditional role in buying food and preparing meals. It also seems possible that an association between dietary factors and cancer risk was missed in men because men underwent greater dietary changes following retirement compared with women and thus we were unable to capture the 'true' exposure status in men (i.e., accurate dietary status during the most relevant time period preceding diagnosis). However, the average age of subjects at entry was 74 years so that the majority had been retired already for nearly 10 years assuming an age of retirement was 65. Although unlikely, it should also be noted that the observed differences in relative risk estimate may be due to the fact that tertiles were created for males and females, separately, and thus represent different absolute intake levels.

The protective effects of the several food groups observed in women might be explained at least in part by the high content of vitamin C in those foods because both dietary vitamin C intake and vitamin C supplement use showed a protective effect. A protective effect on colon cancer risk in women was also observed for total vitamin C intake. Previous evidence for a protective role of vitamin C against colorectal cancer is far from conclusive although some studies have supported the hypothesis (Chen & Barnes, 1990). In a case-control study of Chinese, Whittemore *et al.* found an inverse relation between frequency of vegetable consumption and risk of colorectal cancer but frequency of fruit intake had no effect (Whittemore *et al.*, 1990). Willett *et al.* (1990*b*) reported an inverse association between fruit fibre intake and colon cancer risk in the Nurses Health Study, but neither that effect nor the effect of vitamin C intake were statistically significant after total energy intake and consumption of fat were considered.

Dietary fibre is another candidate nutrient that may explain the protective effect of vegetables and fruits on colon cancer risk (Lanza & Greenwald, 1989). However, the database of dietary fibre content in various foods is still provisional (Lanza & Butrum, 1986) and our questionnaire was not devised to measure dietary fibre intake specifically.

In our study, since total energy intake and fat intake were not evaluated, adjustment for these factors could not be made. However, adjustment for body mass index or physical activity did not materially alter the results (data not shown).

Incidence rates of cancer observed in this largely white population are generally comparable with national figures for whites (Muir *et al.*, 1987) with the exception of a low observed incidence of lung cancer (an expected result, however, based on the low smoking rate in this cohort). It is unlikely, therefore, that a large proportion of incident cases of cancer were missed, which could have led to biased results. The high follow-up rate in terms of vital status (virtually 100%) also supports the completeness of case detection.

Accurate dietary assessment of individuals has been of major interest to investigators trying to explore the assocation between diet and cancer. Several dietary methods have been compared (Gray *et al.*, 1984; Willett, 1990*c*). Jain *et al.* (1980) reported that the dietary history method showed sufficient validity and reliability to make it a useful instrument for epidemiologic studies. The food frequency questionnaire method has advantages over short-term dietary methods such as 24 h recall and dietary records in large scale epidemiologic studies (Morgan *et al.*, 1978). Although quantitative data on portion size of foods increase accuracy in estimating the intake of specific nutrients (Hankin, 1987). Samet *et al.* (1984) noted that portion-size questions provided little additional information in ranking individuals. Humble *et al.* (1987) reported that vitamin A intake calculated with and without portion sizes was similarly related to a reduced risk of lung cancer.

In the present study, we did not collect information on portion size for each food item. Although the composite scores may not be proportional to the absolute intake of nutrients such as vitamins and fibre, we expect the scores to reflect the relative distribution of vegetable and fruit intake among the study population and to serve the purpose of ranking subjects with regard to consumption of those nutrients. We previously reported the results of a comparison of two other methods (index based on intake frequencies and stepwise multiple regression) to estimate vitamin A and C intakes in 50 of these subjects, with those obtained from this self-administered food frequency questionnaire (Gray *et al.*, 1984). The three methods generally provided similar results in terms of broad classification categories for these two nutrients.

The difference in median intake of beta-carotene between 'high' and 'low' tertiles in this study was about 5-fold. Such dietary differences have been shown to be associated with substantial differences in serum levels of beta-carotene. There was also a substantial range in median intake between the high and low tertile categories for vitamin C intake and for the various food groups assessed in this study. However, the estimated daily beta-carotene intake for 9% of men and 56% of women in the lowest tertile was above the US Recommended Dietary Allowance of vitamin A. Although nutrient intakes calculated from food frequency questionnaires tend to be overestimates of actual consumption, the population under study here seems to have relatively high consumption of beta-carotene (and presumably other micronutrients as well) on the average. In fact, the average intake of the foods and nutrients of interest and of vitamin supplements was likely to be sufficiently high so as to preclude detection of a potential harmful effect of very low intake of these food groups or nutrients on cancer risk.

If micronutrients can reduce cancer risk, it is uncertain what the critical period in which dietary factors would play a protective role might be. We presume, perhaps erroneously, that a dietary assessment taken late in life provides a reasonable assessment of usual adult diet. In our study, we captured the average dietary pattern in the 12 months before the questionnaire was administered. It is possible that undetected cancer might have affected dietary patterns of the subjects. However, in the present analysis, we exluded cancer cases diagnosed during the first year of follow-up. In addition, the statistically significant findings for colon cancer observed among women were very similar between the first 3 years of follow-up and the more recent follow-up period (data not shown).

Uncertainties about the exposure variables, as discussed above, might have caused misclassification of subjects. As the data on diet were collected before the cancers developed, the misclassification would be non-differential. Such misclassification could have diluted the effects, if any, of the dietary variables and the specific micronutrients under investigation on cancer incidence. Nonetheless, we interpret our data as providing no strong support for a protective effect of any of the food groups, of vitamin supplements, or of dietary beta-carotene intake on cancer risk.

This study was supported by US Public Health Service grants CA-17054 and CA-32197 from the National Cancer Institute. The authors gratefully acknowledge the invaluable assistance of their research staff – Beverly Ducey, Marian Hawk, and Grace Hsu – and are indebted to the residents of Leisure World, Laguna Hills, whose cooperation made this work possible.

### References

AMES, B.N. (1983). Dietary carcinogens and anticarcinogens. *Science*, **221**, 1256–1264.

AOKI, K., ITO, Y., SASAKI, R., OHTANI, M., HAMAJIMA, N. & ASANO, A. (1987). Smoking, alcohol drinking and serum carotenoids levels. *Jpn. J. Cancer Res.*, **78**, 1049–1056.

BAKER, R.J. & NELDER, J.A. (1978). The GLIM system: release 3. Oxford: Numerical Algorithms Group.

BURING, J.E. & HENNEKENS, C.H. (1989). The possible role of beta-carotene in cancer prevention. *Cancer Prevention*, 1–9.

CHEN, L.H. & BARNES, K.J. (1990). The value of vitamin C in preventing cancer. *Cancer Prevention*, 1-16.

COLEMAN, M., DOUGLAS, A. & HERMON, C. (1985). *User Manual for MANYEARS*. Oxford: Imperial Cancer Research Fund.

DOLL, R. & PETO, R. (1981). *The Causes of Cancer*. Oxford: Oxford University Press.

FONTHAM, E.T.H., PICKLE, L.W., HAENSZEL, W., CORREA, P., LIN, Y. & FALK, R.T. (1988). Dietary vitamins A and C and lung cancer risk in Louisiana. *Cancer*, **62**, 2267-2273.

FONTHAM, E.H. (1990). Protective dietary factors and lung cancer. *Int. J. Epidemiol.*, **19**, S32-S42.

GEY, K.F., BRUBACHER, G.B. & STAHELIN, H.B. (1987). Plasma levels of antioxidant vitamins in relations to ischemic heart disease and cancer. *Am. J. Clin. Nutr.*, **45**, 1368-1377.

GRAHAM, S., DAYAL, H., SWANSON, M., MITTELMAN, A. & WILKINSON, G. (1978). Diet in the epidemiology of cancer of the colon and rectum. *J. Natl Cancer Inst.*, **61**, 709-714.

GRAHAM, S., HAUGHEY, B., MARSHALL, J., PRIORE, R., BYERS, T., RZEPKA, T., METTLIN, C. & PONTES, E. (1983). Diet in the epidemiology of carcinoma of the prostate gland. *J. Natl Cancer Inst.*, **70**, 687-692.

GRAHAM, S., MARSHALL, J., METTLIN, C., RZEPKA, T., NEMOTO, T. & BYERS, T. (1982). Diet in the epidemiology of breast cancer. *Am. J. Epidemiol.*, **116**, 68-75.

GRAY, G.E., PAGANINI-HILL, A., ROSS, R.K. & HENDERSON, B.E. (1984). Assessment of three brief methods of estimation of vitamin A and C intake for a prospective study of cancer: comparison with dietary history. *Am. J. Epidemiol.*, **119**, 581-590.

HANKIN, J.H. (1987). Dietary methods for estimating vitamin A and carotene intakes in epidemiologic studies of cancer. *J. Canadian Dietet. Assoc.*, **48**, 219-224.

HIRAYAMA, T. (1979). Diet and cancer. *Nutr. Cancer*, **1**, 67-81.

HSING, A.W., COMSTOCK, G.W., ABBEY, H. & POLK, B.F. (1990). Serologic precursors of cancer: retinol, carotenoids, and tocopherol and risk of prostate cancer. *J. Natl Cancer Inst.*, **82**, 941-946.

HUMBLE, C.G., SAMET, J.M. & SKIPPER, B.E. (1987). Use of quantified and frequency indices of vitamin A intake in a case-control study of lung cancer. *Int. J. Epidemiol.*, **16**, 341-346.

JAIN, M., HOWE, G.R., JOHNSON, K.C. & MILLER, A.B. (1980). Evaluation of a diet history questionnaire for epidemiologic studies. *Am. J. Epidemiol.*, **111**, 212-219.

KERGOAT, M.J., LECLERC, B.S., PETITCLERC, C. & IMBACH, A. (1988). Determinants of total serum carotene concentration in institutionalized elderly. *J. Am. Geriatr. Soc.*, **36**, 430-436.

KOLONEL, L.N., YOSHIZAWA, C.N. & HANKIN, J.H. (1988). Diet and prostatic cancer: a case-control study in Hawaii. *Am. J. Epidemiol.*, **127**, 999-1012.

LANZA, E. & BUTRUM, R. (1986). A critical review of food fiber analysis and data. *J. Am. Diet. Assoc.*, **86**, 732-740.

LANZA, E. & GREENWALD, P. (1989). The role of dietary fiber in cancer prevention. *Cancer Prevention*, 1-9.

LEMARCHAND, L., YOSHIZAWA, C.N., KOLONEL, L.N., HANKIN, J.H. & GOODMAN, M.T. (1989). Vegetable consumption and lung cancer risk: a population-based case-control study in Hawaii. *J. Natl Cancer Inst.*, **81**, 1158-1164.

MACLENNAN, R. & DECOSTA, J. (1977). Risk factors for lung cancer in Singapore Chinese, a population with high female incidence rates. *Int. J. Cancer*, **20**, 854-860.

MENKES, M.S., COMSTOCK, G.W. & VUILLEUMIER, J.P. (1986). Serum beta-carotene, vitamins A and E, selenium, and the risk of lung cancer. *N. Engl. J. Med.*, **315**, 1250-1254.

METTLIN, C., GRAHAM, S. & SWANSON, M. (1979). Vitamin A and lung cancer. *J. Natl Cancer Inst.*, **62**, 1435-1438.

MORGAN, R.W., JAIN, M., MILLER, A.B., CHOI, N.W., MATTHEWS, V., MUNAN, L., BURCH, J.D., FEATHER, J., HOWE, G.R. & KELLY, A. (1978). A comparison of dietary methods in epidemiologic studies. *Am. J. Epidemiol.*, **107**, 488-498.

MUIR, C., WATERHOUSE, J., MACK, T., POWELL, J. & WHELAN, S. (eds) (1987). *Cancer Incidence in Five Continents* (volume V). Lyon: IARC.

NOMURA, A.M., STEMMERMANN, G.N., HEILBRUN, L.K., SALKELD, R.M. & VUILLEUMIER, J.P. (1985). Serum vitamin levels and risk of cancer of specific sites in men of Japanese ancestry in Hawaii. *Cancer Res.*, **45**, 2369-2372.

OHNO, Y., YOSHIDA, O., OISHI, K., OKADA, K., YAMABE, H. & SCHROEDER, F.H. (1988). Dietary beta-carotene and cancer of the prostate: a case-control study. *Cancer Res.*, **48**, 1331-1336.

PASTORINO, U., PISANI, P., BERRINO, J., ANDREOLI, C., BARBIERI, A., COSTA, A., MAZZOLENI, C., GRAMEGNA, G. & MARUBINI, E. (1987). Vitamin A and female lung cancer: a case-control study on plasma and diet. *Nutr. Cancer*, **10**, 171-179.

PETO, R., DOLL, R., BUCKLEY, J.D. & SPORN, M.B. (1981). Can dietary beta-carotene materially reduce human cancer rates? *Nature*, **290**, 201-208.

RISCH, H.A., BURCH, J.D., MILLER, A.B., HILL, G.B., STEELE, R. & HOWE, G.R. (1988). Dietary factors and the incidence of cancer of the urinary bladder. *Am. J. Epidemiol.*, **127**, 1179-1191.

ROIDT, L., WHITE, E., GOODMAN, G.E., WAHL, P.W., OMENN, G.S., ROLLINS, B. & KARKECK, J.M. (1988). Association of food frequency questionnaire estimates of vitamin A intake with serum vitamin A levels. *Am. J. Epidemiol.*, **128**, 645-654.

RUSSEL-BRIEFEL, R., BATES, M.W. & KULLER, L.H. (1985). The relationship of plasma carotenoids to health and biochemical factors in middle-aged men. *Am. J. Epidemiol.*, **122**, 741-749.

SAMET, J.M., HUMBLE, C.G. & SKIPPER, B.E. (1984). Alternatives in the collection and analysis of food frequency interview data. *Am. J. Epidemiol.*, **120**, 572-581.

SCHOBER, S.E., COMSTOCK, G.W., HELSING, K.J., SALKELD, R.M., MORRIS, J.S., RIDER, A.A. & BROOKMEYER, R. (1987). Serologic precursors of cancer, I. prediagnostic serum nutrients and colon cancer risk. *Am. J. Epidemiol.*, **126**, 1033-1041.

SHEKELLE, R.B., LIU, S., RAYNOR, W.J. Jr., LEPPER, M., MALIZA, C. & ROSSOF, A.H. (1981). Dietary vitamin A and risk of cancer in the Western Electric Study. *Lancet*, **2**, 1185-1189.

SHIBATA, A., SASAKI, R., ITO, Y., HAMAJIMA, N., SUZUKI, S., OHTANI, M. & AOKI, K. (1989). Serum concentration of beta-carotene and intake frequency of green-yellow vegetables among healthy inhabitants of Japan. *Int. J. Cancer*, **44**, 48-52.

STRYKER, W.S., KAPLAN, L.A., STEIN, E.A., STAMPFER, M.J., SOBER, A. & WILLETT, W.C. (1988). The relationship of diet, cigarette smoking, and alcohol consumption to plasma beta-carotene and alpha-tocopherol levels. *Am. J. Epidemiol.*, **127**, 283-296.

U.S. DEPARTMENT OF AGRICULTURE (1976-1984). *Composition of foods* (Handbook Nos. 8.1-8.14). Washington D.C.: US Government Printing Office.

WALD, N.J., BOREHAM, J., HAYWARD, J.L. & BULBROOK, R.D. (1984). Plasma retinol, beta-carotene and vitamin E levels in relation to the future risk of breast cancer. *Br. J. Cancer*, **49**, 321-324.

WALD, N.J., THOMPSON, S.G., DENSEM, J.W., BOREHAM, J. & BAILEY, A. (1988). Serum beta-carotene and subsequent risk of cancer: results from the BUPA Study. *Br. J. Cancer*, **57**, 428-433.

WHITTEMORE, A.S., WU-WILLIAMS, A.H., LEE, M., SHU, Z., GALLGHER, R.P., DENG-AO, J., LUN, Z., XIANGHUI, W., KUN, C., JUNG, D., TEH, C.-Z., CHENGDE, L., YAO, X.J., PAFFENBARGER, R.S. Jr. & HENDERSON, B.E. (1990). Diet, physical activity, and colorectal cancer among Chinese in North America and China. *J. Natl Cancer Inst.*, **82**, 915-926.

WILLETT, W.C., POLK, B.F., UNDERWOOD, B.A., STAMPFER, M.J., PRESSEL, S., ROSNER, B., TAYLOR, J.O., SCHNEIDER, K. & HAMES, C.G. (1984). Relation of serum vitamins A and E and carotenoids to the risk of cancer. *N. Engl. J. Med.*, **310**, 430-434.

WILLET, W.C. (1990*a*). Vitamin A and lung cancer. *Nutr. Rev.*, **48**, 201-211.

WILLETT, W.C., STAMPFER, M.J., GOLDITZ, G.A., ROSNER, B.A. & SPEIZER, F.E. (1990*b*). Relation of meat, fat, and fiber intake to the risk of colon cancer in a prospective study among women. *N. Engl. J. Med.*, **323**, 1664-1672.

WILLETT, W. (1990*c*). Food frequency methods. In *Nutritional Epidemiology*, Willett, W. pp. 69-91. New York: Oxford University Press.

WU, A.H., HENDERSON, B.E., PIKE, M.C. & YU, M.C. (1985). Smoking and other risk factors for lung cancer in women. *J. Natl Cancer Inst.*, **74**, 747-751.

WU, A.H., PAGANINI-HILL, A., ROSS, R.K. & HENDERSON, B.E. (1987). Alcohol, physical activity and other risk factors for colorectal cancer: a prospective study. *Br. J. Cancer*, **55**, 687-694.

ZIEGLER, R.G. (1986). Epidemiologic studies of vitamins and cancer of the lung, oesphagus, and cervix. *Adv. Exp. Med. Biol.*, **206**, 11-26.

### APPENDIX

Food items included in the questionnaire [portion sizes utilised for calculation of beta-carotene and vitamin C intakes]

*Vegetables*

Leafy green lettuce (Romaine, Boston, bibb, butterhead, endive, escarole, salad bowl, red leafy lettuce) [1 cup]
*Other leafy greens (spinach, chard, beet greens, turnip greens, mustard greens, collards, kale, dandelion greens) [½ cup cooked and 1 cup raw]
Iceberg or head lettuce [1 cup]
Cabbage (include sauerkraut and coleslaw) [1 cup]
White potatoes or turnips [1 potato]
**Sweet potatoes, yams, pumpkin (include use in pie or soup) [½ potato]
**Carrots [½ carrot]
Winter squash (butternut, hubbard, acorn squash – include use in pie or soup) [½ cup]
**Summer squash (zucchini, yellow crookneck, yellow straightneck, cocozelle, scallop squash) [½ cup]
*Broccoli [½ cup]
Tomatoes (fresh or cooked, including tomatoes in a sauce such as spaghetti sauce or tomato soup) [1 tomato]
Green peas (include snow peas and Chinese pea pods) [½ cup]
Green beans or string beans [½ cup]
Lima beans or blackeye beans [½ cup]
Corn [1 ear]
Asparagus [4 spears]
Sweet green peppers [½ cup]
**Sweet red peppers [1 pepper]
**Hot red chili peppers (include hot pepper sauce, chili powder, cayenne pepper, tabasco sauce) [1/10 pepper]
*Brussel sprouts [4 sprouts]
Cauliflower [½ cup]

*Fruits*

Cantaloups, mangos [¼ fruit]
Watermelon [1/32 fruit]
Apricots, nectarines (include apricot nectar) [3 apricots or 1 nectarine]
Peaches [1 fruit]
Papayas [1 cup]
Persimmons [1 fruit]
Sour cherries [1 cup]
Prunes, prune juice [5 fruits]
Apples, applesauce (not apple juice) [1 fruit]
Bananas [1 fruit]
Avocados, guacamole [½ cup]
Pineapple, pineapple juice [½ cup]
Blackberries, blueberries, raspberries, boysenberries, loganberries, sweet cherries [½ cup]
Fruit cocktail [½ cup]
Oranges, tangerines, mandarin oranges, orange juice [1 fruit or 1 cup of juice]
White grapefruit and juice [½ fruit]
Pink/red grapefruit and juice [½ fruit]
Honeydew, casaba melons [1/10 fruit]
Strawberries [½ cup]
Cranberry juice cocktail [1 cup]
Plums [1 fruit]
Rhubarb [½ cup]
Grapes, pears, figs, raisins, dates [10 grapes, 1 pear, 1 tablespoon of raisins, 1 fig, or 10 dates]

* items included in 'dark green vegetables'
** items included in 'yellow vegetables'